# Genomic and clinical characteristics of carbapenem-resistant *Enterobacter cloacae* complex isolates collected in a Chinese tertiary hospital during 2013–2021

**DOI:** 10.3389/fmicb.2023.1127948

**Published:** 2023-02-21

**Authors:** Mei Han, Chang Liu, Hui Xie, Jie Zheng, Yan Zhang, Chuchu Li, Han Shen, Xiaoli Cao

**Affiliations:** ^1^Department of Laboratory Medicine, Nanjing Drum Tower Hospital, The Affiliated Hospital of Nanjing University Medical School, Nanjing, Jiangsu, China; ^2^Department of Acute Infectious Disease Control and Prevention, Jiangsu Provincial Center for Disease Control and Prevention, Nanjing, Jiangsu, China

**Keywords:** *Enterobacter cloacae* complex, carbapenem resistance, whole-genome sequencing, resistant determinants, sequence types, plasmid replicons, clinical characteristics

## Abstract

**Objective:**

To analyze the molecular epidemiology of carbapenem-resistant *Enterobacter cloacae* complex (CREC) by whole-genome sequencing and to explore its clinical characteristics.

**Methods:**

*Enterobacter cloacae* complex isolates collected in a tertiary hospital during 2013–2021 were subjected to whole-genome sequencing to determine the distribution of antimicrobial resistance genes (ARGs), sequence types (STs), and plasmid replicons. A phylogenetic tree of the CREC strains was constructed based on the whole-genome sequences to analyze their relationships. Clinical patient information was collected for risk factor analysis.

**Results:**

Among the 51 CREC strains collected, *bla*NDM-1 (*n* = 42, 82.4%) was the main carbapenem-hydrolyzing β-lactamase (CHβL), followed by *bla*IMP-4 (*n* = 11, 21.6%). Several other extended-spectrum β-lactamase-encoding genes were also identified, with *bla*SHV-12 (*n* = 30, 58.8%) and *bla*TEM-1B (*n* = 24, 47.1%) being the predominant ones. Multi-locus sequence typing revealed 25 distinct STs, and ST418 (*n* = 12, 23.5%) was the predominant clone. Plasmid analysis identified 15 types of plasmid replicons, among which IncHI2 (*n* = 33, 64.7%) and IncHI2A (*n* = 33, 64.7%) were the main ones. Risk factor analysis showed that intensive care unit (ICU) admission, autoimmune disease, pulmonary infection, and previous corticosteroid use within 1 month were major risk factors for acquiring CREC. Logistic regression analysis showed that ICU admission was an independent risk factor for CREC acquisition and was closely related with acquiring infection by CREC with ST418.

**Conclusion:**

*Bla*NDM-1 and *bla*IMP-4 were the predominant carbapenem resistance genes. ST418 carrying *Bla*NDM-1 not only was the main clone, but also circulated in the ICU of our hospital during 2019–2021, which highlights the necessity for surveillance of this strain in the ICU. Furthermore, patients with risk factors for CREC acquisition, including ICU admission, autoimmune disease, pulmonary infection, and previous corticosteroid use within 1 month, need to be closely monitored for CREC infection.

## 1. Introduction

*Enterobacter cloacae* complex (ECC), which belongs to the family *Enterobacteriaceae*, is a common nosocomial pathogen responsible for several human infections, including bacteremia and respiratory tract, wound, and urinary tract infections (Maria et al., 2012; Jin et al., 2020). Currently, ECC includes *Enterobacter cloacae*, *Enterobacter hormaechei*, *Enterobacter kobei*, *Enterobacter asburiae*, *Enterobacter cancerogenus*, *Enterobacter nimipressuralis*, and *Enterobacter mori*. *E. cloacae* and *E. hormaechei* are the most frequently isolated from human clinical specimens (Sutton et al., 2018; Davin-Regli et al., 2019).

Carbapenems have been extensively used in clinic as a last choice for treating severe gram-negative bacterial infection owing to their broad-spectrum antibacterial activity and non-toxicity (Jin et al., 2018). In recent years, with the widespread use of carbapenem antibiotics, the emergence and spread of carbapenem-resistant *Enterobacteriaceae* (CRE) has posed a serious public health threat (Davin-Regli and Pages, 2015; Jiang et al., 2022). At present, carbapenem-resistant *Enterobacter cloacae* complex (CREC) is the third most common CRE following *Escherichia coli* and *Klebsiella pneumoniae* in China (Zhang et al., 2018; Hu et al., 2022). An increased incidence of CREC with clonal transmission and endemicity has been constantly reported in various regions (Jiang et al., 2022).

The main mechanism of carbapenem resistance in CREC is the production of carbapenem-hydrolyzing β-lactamases (CHβLs), among which KPC, NDM, VIM, IMP, and OXA-48 are the most prevalent ones. In addition, overexpression of the β-lactamase AmpC, the occurrence of extended-spectrum β-lactamases (ESBLs), such as TEM, CTX-M, and SHV, membrane-associated mechanisms, such as outer membrane permeability, and the overexpression of efflux pumps have been demonstrated to contribute to carbapenem resistance in ECC (Davin-Regli et al., 2019). The acquisition of the carbapenem resistance genes are most often transferred by mobile genetic elements (MGEs), such as plasmids and transposons, which can be easily transferred between different species (Dong et al., 2022). Globally, NDM is the main CHβL conferring carbapenem resistance in CREC. However, regional differences in the distribution of CREC CHβLs have been observed, with KPC being predominant in North America, OXA-48 and VIM being predominant in Europe, and NDM being the most prevalent in China (Annavajhala et al., 2019).

A multinational surveillance study based on multi-locus sequence typing (MLST) revealed substantial clonal diversity of CREC, and ST114, ST93, ST90, and ST78 were potential high-risk clones globally widespread in 37 countries (Peirano et al., 2018). Risk factor analysis for CREC acquisition has shown that ICU admission, prior antimicrobial exposure, invasive treatments, surgical procedures, and prolonged hospitalization are the main risk factors for CREC acquisition (Jiang et al., 2019; Li et al., 2019; Tetsuka et al., 2019; Tian et al., 2020). However, studies on a long-term continuous study of CREC has been poorly reported in China.

The present study was performed in Nanjing Drum Tower Hospital, a 3800-bed tertiary hospital. A total of 51 CREC strains collected during 2013–2021 were subjected to whole-genome sequencing (WGS) to analyze the distribution of antimicrobial resistance genes (ARGs), sequence types (STs), and plasmid replicons. Clinical patient information was collected to analyze risk factors for CREC acquisition. We attempted to systematically analyze the molecular epidemiology and clinical characteristics of CREC infection to provide evidence for effective control of nosocomial infection with CREC.

## 2. Materials and methods

### 2.1. Bacterial isolates

In total, 51 consecutive, non-duplicate CREC isolates were collected in Nanjing Drum Tower Hospital (the affiliated hospital of Nanjing University Medical School) during 2013–2021. All the strains were verified by Vitek 2.0 (BioMérieux, Marcy- l’Étoile, France) and matrix-associated laser desorption ionization–time of flight mass spectrometry (MALDI-TOF MS) (BioMérieux, Craponne, France). And these strains were confirmed as CREC by testing the susceptibilities to imipenem by Bauer et al. (1966) method according to the Clinical and Laboratory Standards Institute (CLSI) criteria (Humphries et al., 2021).

The strains were isolated from the following samples: sputum (*n* = 16, 31.4%), abdominal dropsy (*n* = 8, 15.7%), blood (*n* = 8, 15.7%) secretion (*n* = 7, 13.7%), urine (*n* = 6, 11.8%), catheter (*n* = 3, 5.9%), pleural effusion (*n* = 2, 3.9%), and bronchial alveolar lavage fluid (*n* = 1, 2.0%). The numbers of strains isolated in each year were as follows: 2013 (*n* = 1, 2.0%), 2014 (*n* = 2, 3.9%), 2015 (*n* = 4, 7.8%), 2016 (*n* = 2, 3.9%), 2017 (*n* = 5, 9.8%), 2018 (*n* = 7, 13.7%), 2019 (*n* = 8, 15.7%), 2020 (*n* = 9, 17.6%), and 2021 (*n* = 13, 25.5%). The CREC isolates were mostly collected from the ICU (*n* = 16, 30.8%), surgical ward (*n* = 7, 13.5%), cardiac surgery ward (*n* = 3, 5.8%), hematology ward (*n* = 3, 5.8%), and respiratory ward (*n* = 3, 5.8%).

### 2.2. Genomic DNA extraction, whole-genome sequencing, *de novo* assembly, scaffolding, and annotation

Fresh colonies of 51 CREC strains were picked from Luria–Bertani plates (Oxoid, UK) for genomic DNA extraction using the Ultraclean Microbial DNA Isolation Kit (MO BIO Laboratories, Carlsbad, CA, USA) according to the manufacturer’s recommendations. After a quality check, the genomic DNA samples were sent to Beijing Tiangen Biochemical Technology (Beijing, China) for sequencing on the MiSeq platform (Illumina, San Diego, CA, USA). After quality trimming (Qs ≥ 20), *de novo* assembly of the paired-end reads was performed using CLC Genomics Workbench v21.0.4 (Qiagen, Hilden, Germany). Scaffolding was performed using SSPACE standard version 3.0 and gaps within scaffolds were closed using GapFiller (Boetzer et al., 2011). The assembled genomes were submitted to NCBI for annotation.

### 2.3. Analysis of genomic epidemiology

Genomes of 51 CREC isolates have been uploaded to the NCBI BioProject repository^1^ with the accession number PRJNA868583 and are publicly available. MLST of strains retrieved from GenBank was performed using MLST v2.0.^2^ ARGs were identified using Resfinder v2.1.^3^ Antibiotic resistance plasmid genes were analyzed by uploading the genomes to PlasmidFinder v2.1.^4^

### 2.4. Phylogenetic analysis

The genome sequences of *E. hormaechei* strains were annotated using Prokka v1.14.6 (Seemann, 2014). The gff files produced by Prokka were used as input for genomic analysis using Roary v3.13.0, and SNP alignment sequences of 3,135 core genes were obtained (Page et al., 2015). The nucleotide substitution model was determined to be general time reversal model using jModelTest v2.0 (Darriba et al., 2012). A maximum-likelihood evolutionary tree was constructed using the RAxML-NG software v1.1.0 with bootstrapping set to 1000 (Kozlov et al., 2019) and was visualized using iTOL v6.5.8 Branches with bootstrap values of less than 50 were deleted (Letunic and Bork, 2021).

### 2.5. Clinical information collection

Among the 51 patients with CREC isolates, the clinical information of 50 ones were available to identify possible risk factors for CREC acquisition, whereas, clinical information of one patient with CREC was missing. Meanwhile, fifty isolates from patients with carbapenem-susceptible *Enterobacter cloacae* (CSEC) collected during the same time period and in the same wards and anatomical sites were included as a control group. The following factors were analyzed: patient demographics, underlying diseases and acquired infections, invasive procedures during hospital stay, and treatments within 1 month (Tetsuka et al., 2019; Tian et al., 2020; Oka et al., 2022). The study was approved by the Ethics Committee of Nanjing Drum Tower Hospital (approval No. 2022-049-2) and dispensed the need to obtain signed informed consents on account of the absence of any intervention.

### 2.6. Statistical analysis

The SPSS software (v25.0) was conducted for statistical analysis. The results of univariate analysis were reported as *P*-values by using the Chi-square test. To identify independent risk factors for CREC acquisition, significant variables with *P* < 0.10 in univariate analysis were enrolled in the logistic regression model for multivariate analysis (Tian et al., 2020). *P* < 0.05 was considered statistically significant. McNemar-Bowker test was used to analyze the distribution consistency between ARGs and plasmid replicons. *P*-value > 0.05 was taken as the consistency between ARGs and plasmid replicons.

## 3. Results

### 3.1. ECC species identified

**FIGURE 1:**
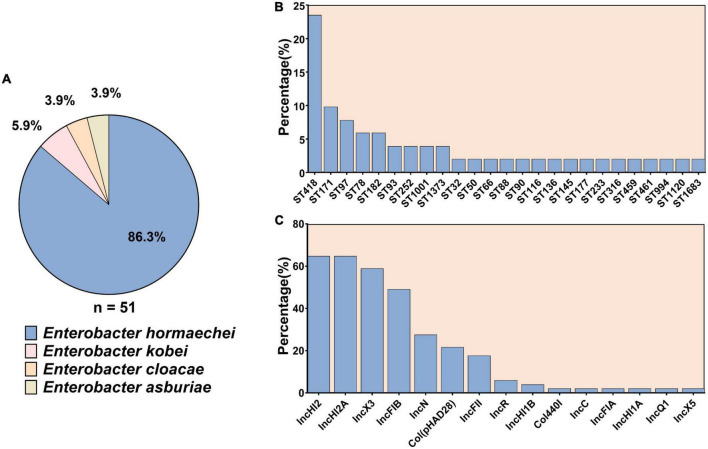
The subspecies and sequence types of 51 carbapenem-resistant *Enterobacter cloacae* complex as well as the distribution of plasmid replicons among them. **(A)** The subspecies of 51 carbapenem-resistant *Enterobacter cloacae* complex. **(B)** Sequence types of the 51 carbapenem-resistant *Enterobacter cloacae* complex strains. **(C)** Plasmid replicons among the 51 carbapenem-resistant *Enterobacter cloacae* complex.

Among the 51 ECC isolates, 44 (86.3%) isolates were identified as *Enterobacter hormaechei*, three (5.9%) as *Enterobacter kobei*, two (3.9%) as *Enterobacter cloacae*, and the two (3.9%) as *Enterobacter asburiae* (Figure 1A).

### 3.2. Distribution of antimicrobial resistance genes

**FIGURE 2:**
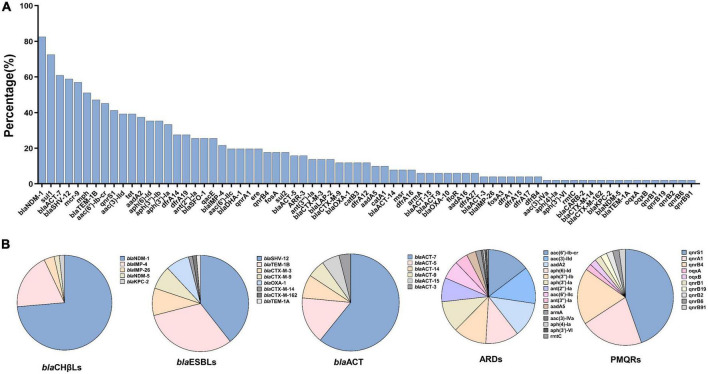
Antimicrobial resistance genes and the detailed subtypes detected among 51 carbapenem-resistant *Enterobacter cloacae* complex isolates. **(A)** Distribution of antimicrobial resistance genes ranked by the percentages. **(B)** The detailed subtypes of some predominant resistance genes. *bla*CHβLs, carbapenem-hydrolyzing β-lactamases; *bla*ESBLs, extended-spectrum β-lactamases; ARDs, aminoglycoside resistant determinants; PMQRs, plasmid mediated quinolone resistance genes.

Various genes conferring resistance to 12 antimicrobial agents were identified in the 51 CREC isolates (Figure 2).

Five *bla*CHβls were identified; *bla*NDM-1 (*n* = 42, 82.4%) and *bla*IMP-4 (*n* = 11, 21.6%) were the predominant ones, followed by *bla*IMP-26 (*n* = 2, 3.9%), *bla*NDM-5 (*n* = 1, 2.0%), and *bla*KPC-2 (*n* = 1, 2.0%). Of note, co-existence of *bla*NDM-1 and *bla*IMP-4 was detected in six isolates (EC6949, EC13079, EC13224, EC13343, EC15480, and EC16542). Co-existence of *bla*NDM-1 and *bla*KPC-2 was detected in one isolate, EC13565. As for *bla*ESBLs, *bla*SHV-12 (*n* = 30, 58.8%), *bla*TEM-1B (*n* = 24, 47.1%), *bla*CTX-M-3 (7, 13.7%), *bla*CTX-M-9 (*n* = 6, 11.8%), *bla*OXA-1 (*n* = 6, 11.8%) and *bla*CTX-M-14, *bla*CTX-M-162, and *bla*TEM-1A (*n* = 1, 2.0% each) were identified (Figure 2B).

All 51 ECC isolates carried *bla*ACT with *bla*ACT-7 being the predominant one (Figure 2). Of note, mobile colistin resistance (*mcr*)-9 (29/51, 56.9%) was highly prevalent among the CREC isolates in this study (Figure 2A). Co-existence of *mcr-9* and *bla*NDM-1, *bla*IMP-4, *bla*IMP-26, or KPC-2 was identified in more than 50% of the CREC isolates (Supplementary Table 1).

### 3.3. STs of the 51 CREC strains

The 51 CREC isolates were assigned to 25 STs. ST418 (12/51, 23.5%) was the most prevalent, followed by ST171 (5/51, 9.8%), ST97 (4/51, 7.8%), ST78 (3/51, 5.9%), ST182 (3/51, 5.9%), ST93 (2/51, 3.9%), ST252 (2/51, 3.9%), ST1001 (2/51, 3.9%), and ST1373 (2/51, 3.9%). The remaining STs were only detected in one isolate (Figure 1B).

### 3.4. Plasmid replicons in the 51 CREC strains

Fifteen types of plasmid replicons were identified in 98% (50/51) of the isolates: IncHI2 (*n* = 33, 64.7%) and IncHI2A (*n* = 33, 64.7%) were the most prevalent ones, followed by IncX3 (*n* = 30, 58.8%), IncFIB (*n* = 25, 49.0%), IncN (*n* = 14, 27.5%), and Col (pHAD28) (*n* = 11, 21.6%). The remaining plasmid replicons were relatively less common (Figure 1C). No plasmid replicons were found in isolate EC15999.

Statistical analysis on the distribution consistency between ARGs and plasmid replicons in the CREC isolates are shown in Table 1. There was a consistency in the distribution of *bla*IMP and IncN, and the consistent distribution was also observed between *bla*SHV-12 and IncHI2, IncHI2A, IncX3, or IncFIB. Moreover, consistency distribution between *mcr-9* and IncHI2, IncHI2A, IncX3, or IncFIB was also found.

**TABLE 1:** Distribution of plasmid replicons and antimicrobial resistance genes in the 51 carbapenem-resistant *Enterobacter cloacae* complex (CREC) isolates.

	IncHI2 (*n* = 33)	IncHI2A (*n* = 33)	IncX3 (*n* = 30)	IncFIB (*n* = 25)	IncN (*n* = 14)
*bla*NDM (*n* = 43)	0.031	0.031	0.000	0.001	0.000
*bla*IMP (*n* = 13)	0.000	0.000	0.003	0.008	**1.000**
*bla*SHV-12 (*n* = 30)	**0.648**	**0.648**	**1.000**	**0.332**	0.007
*mcr*-9 (*n* = 29)	**0.289**	**0.289**	**1.000**	**0.541**	0.004

Bold values indicate *p*-value of >0.05 was taken as the consistency between antimicrobial resistance genes and plasmid replicons.

### 3.5. Phylogeny of *E. hormaechei*

**FIGURE 3:**
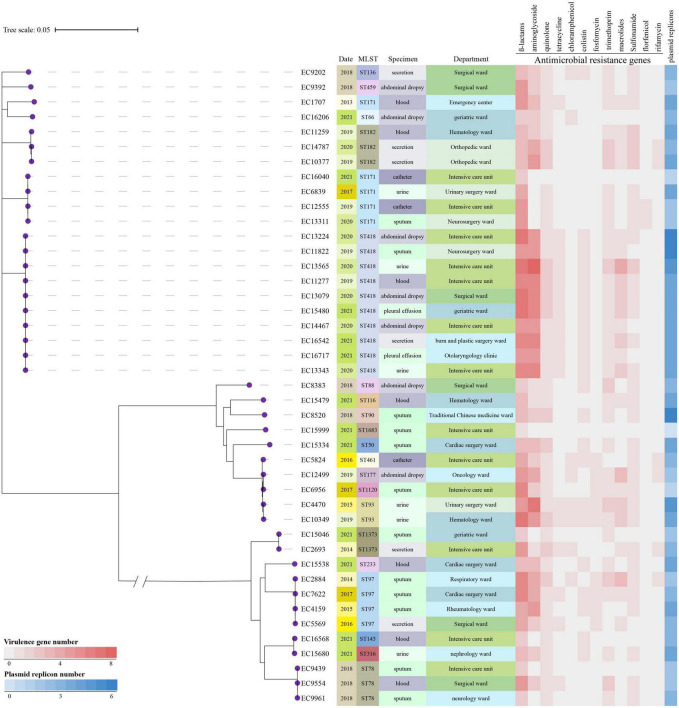
Phylogenetic tree and heatmap of the 44 *Enterobacter hormaechei* isolates. Phylogenetic tree was constructed based on single nucleotide polymorphism alignment of genomes. MLST, multi-locus sequence typing.

The phylogenetic tree of the 44 *E. hormaechei* isolates displayed genetic diversity, and 17 clades were distinguished (Figure 3). Ten ST418 clones formed the largest clade, indicating clonal dissemination of ST418 strains in our hospital during 2019–2021. In addition, we found clonal dissemination of ST97, ST78, and ST171 clones.

### 3.6. Risk factor analysis

The results of the comparison of clinical variables between the CREC and CSEC group are presented in Table 2. Intensive care unit (ICU) admission, autoimmune disease, pulmonary infection, and previous corticosteroid use within 1 month were the main risk factors for CREC acquisition. Multivariate analysis revealed that ICU admission (*P* = 0.008) was an independent risk factor for CREC acquisition (Table 3). The results shown in Table 4 indicate that ICU admission was significantly (*P* = 0.045) correlated with CREC acquisition with ST418 as compared to CREC acquisition with non-ST418 strains.

**TABLE 2:** Univariate analysis of the risk factors for acquiring carbapenem-susceptible *Enterobacter cloacae* complex (CSEC) and carbapenem-resistant *Enterobacter cloacae* complex (CREC).

Variable	CSEC (*n* = 50)		CREC (*n* = 50)		Total	*P*-value
	Count	Percentage	Count	Percentage		
**Demographics**						
Male sex	30	60.0%	37	74.0%	67	0.137
Age ≥ 65 years	24	48.0%	27	54.0%	51	0.548
Admission to ICU	12	24.0%	27	54.0%	39	**0.002**
Clinical outcomes (Die)	3	6.0%	7	14.0%	10	0.317
Prior hospitalization	24	48.0%	27	54.0%	51	0.548
**Baseline diseases and acquired infections**						
Hypertension	21	42.0%	20	40.0%	41	0.839
Diabetes mellitus	9	18.0%	13	26.0%	22	0.334
Autoimmune disease	1	2.0%	9	18.0%	10	**0.020**
Malignancy	20	40.0%	18	36.0%	38	0.680
Agranulocytosis	1	2.0%	2	4.0%	3	1.000
Cerebrovascular disease	12	24.0%	17	34.0%	29	0.271
Cardiovascular disease	16	32.0%	20	40.0%	36	0.405
Kidney disease	8	16.0%	11	22.0%	19	0.444
Chronic respiratory disease	3	6.0%	9	18.0%	12	0.124
Pulmonary infection	15	30.0%	28	56.0%	43	**0.009**
Urinary tract infection	6	12.0%	8	16.0%	14	0.564
Biliary tract infection	9	18.0%	6	12.0%	15	0.401
**Invasive procedures during hospital stay**						
Invasive procedures	40	80.0%	39	78.0%	79	0.806
Invasive surgery	34	68.0%	28	56.0%	62	0.216
Blood transfusion	22	44.0%	24	48.0%	46	0.688
**Treatments within 1 month**						
Previous corticosteroids	5	10.0%	13	26.0%	18	**0.037**
Chemotherapy	2	4.0%	4	8.0%	6	0.674
Prior history of antibiotics	49	98.0%	49	98.0%	98	1.000

ICU, intensive care unit. Bold values indicate significant (*P* < 0.05).

**TABLE 3:** Multivariate analysis of risk factors for carbapenem-susceptible *Enterobacter cloacae* complex (CSEC) and carbapenem-resistant *Enterobacter cloacae* complex (CREC).

Variables	B	S.E	Wals	95% CI	*P*-value
Admission to ICU	−1.221	0.462	6.979	0.119–0.730	**0.008**
Autoimmune disease	−1.765	1.219	2.095	0.016–1.868	0.148
Pulmonary infection	−0.655	0.462	2.014	0.210–1.284	0.156
Previous corticosteroids	−0.484	0.711	0.463	0.153–2.484	0.496

ICU, intensive care unit. Bold values indicate significant (*P* < 0.05).

**TABLE 4:** Univariate analysis of risk factors for infection with ST418 and non-ST418 strains.

Variable	Non-ST418 (*n* = 38)		ST418 (*n* = 12)		Total	*P*-value
	Count	Percentage	Count	Percentage		
**Demographics**						
Male sex	30	78.9%	7	58.3%	37	0.156
Age ≥ 65 years	21	55.3%	6	50.0%	27	0.750
Admission to ICU	17	44.7%	10	83.3%	27	**0.045**
Clinical outcomes (Die)	6	15.8%	1	8.3%	7	0.864
Prior hospitalization	21	55.3%	6	50.0%	27	0.750
**Baseline diseases and acquired infections**						
Hypertension	13	34.2%	7	58.3%	20	0.137
Diabetes mellitus	10	26.3%	3	25.0%	13	1.000
Autoimmune disease	8	21.1%	1	8.3%	9	0.569
Malignancy	14	36.8%	4	33.3%	18	1.000
Agranulocytosis	2	5.3%	0	0.0%	2	1.000
Cerebrovascular disease	12	31.6%	5	41.7%	17	0.520
Cardiovascular disease	15	39.5%	5	41.7%	20	0.892
Kidney disease	8	21.1%	3	25.0%	11	1.000
Chronic respiratory disease	6	15.8%	3	25.0%	9	0.769
Pulmonary infection	20	52.6%	8	66.7%	28	0.603
Urinary tract infection	6	15.8%	2	16.7%	8	1.000
Biliary tract infection	3	7.9%	3	25.0%	6	0.280
**Invasive procedures during hospital stay**						
Invasive procedures	28	73.7%	11	91.7%	39	0.362
Invasive surgery	19	50.0%	9	75.0%	28	0.235
Blood transfusion	18	47.4%	6	50.0%	24	0.874
**Treatments within 1 month**						
Previous corticosteroids	11	28.9%	2	16.7%	13	0.640
Chemotherapy	4	10.5%	0	0.0%	4	0.560
Prior history of antibiotics	37	97.4%	12	100.0%	49	1.000

ICU, intensive care unit. Bold values indicate significant (*P* < 0.05).

## 4. Discussion

The wide spread of the CREC poses a serious public health threat. Therefore, it is urgent to characterize the molecular epidemiology of CREC and risk factors for CREC acquisition to provide more information for guiding the formulation and implementation of infection control measures. In this study, we used WGS technology to analyze the genomic characteristics of clinical CREC isolates collected over a 9-year period in a tertiary hospital in Southeast China. To the best of our knowledge, this is the first study to analyze the genomic epidemiology and potential risk factors for acquisition of CREC isolates collected in Jiangsu Province, China.

CHβLs have increasingly spread globally over the last decades (Poirel et al., 2007). In our study, *bla*NDM-1 was the predominant *bla*CHβL, which is in accordance with the results of a multicenter study conducted in 11 Chinese cities that provided evidence that *bla*NDM-1 is the main CHβL conferring resistance to carbapenem (Zhao et al., 2019), although we also identified *bla*IMP as a major *bla*CHβL. Both *bla*NDM and *bla*IMP are widely disseminated among bacteria (Tzouvelekis et al., 2012). *Bla*IMP-4 is frequently detected in ECC strains, whereas *bla*IMP-26 has been rarely reported in ECC strains (Jin et al., 2020) since it was first reported in a clinical carbapenem-resistant *Pseudomonas aeruginosa* isolate in Singapore in 2010 (Koh et al., 2010). Notably, five *E. hormaechei* isolates co-carrying *bla*NDM-1 and *bla*IMP-4 were found in the clonally disseminated clone ST418, indicating that infection control measures should be implemented to prevent further spread. No strains were found to carry *bla*VIM-1, which is the most common *bla*CHβL in Southern European countries (Villa et al., 2014). *Bla*SHV-12 and *bla*TEM-1B were the predominant *bla*ESBLs in our study which may result from the co-occurrence of these genes among the same mobile elements such as plasmids or integron, whereas CTX-M-producers are the most frequent in Latin American countries (Seki et al., 2013). Of note, all the 51 ECC isolates carried *bla*ACT, which is a plasmid *Amp*C β-lactamase gene widely disseminated among *Enterobacteriaceae*. In addition to the constitutive expression of *Amp*C β-lactamase, the frequent occurrence of *bla*ACT among these strains may lead to decreased susceptibility to cephalosporins (Davin-Regli et al., 2019).

Notably, *mcr-9* was highly prevalent among the CREC isolates in this study. Albeit there was a distribution consistency between *mcr-9* and IncHI2, IncHI2A, IncX3, and IncFIB, *mcr-9* and these plasmids were not in the same contigs among isolates (Supplementary Table 2), so it is difficult to confirm the connection between *mcr-9* and any inc type. Furthermore, as a recently emerging colistin resistance determinants, *mcr-9* was identified firstly in a clinical *Salmonella enterica* isolate in the USA in 2019 (Carroll et al., 2019). Thereafter, the high prevalence of *mcr-9* worldwide has been revealed by ongoing discoveries, posing potential threat to public health (Li et al., 2020). Although isolates harboring *mcr-9* were still susceptible to colistin, active monitoring should be implemented since inducible resistance to colistin has been reported after induction of *mcr-9* expression using colistin (Ling et al., 2020). Co-existence of *mcr-9* and *bla*CHβLs within single CREC isolates as observed in our study has been reported previously. An *E. hormaechei* isolate co-carried *mcr-9* and *bla*VIM-4 or *bla*NDM-1, but on different plasmids (Yuan et al., 2019; Soliman et al., 2020), and a clinic *E. cloacae* complex strain co-harbored *mcr-9* and *bla*IMP-1 (Kananizadeh et al., 2020). In the current study, co-existence of *mcr-9* and *bla*NDM-1, *bla* IMP-4, *bla* IMP-26, or KPC-2 was identified in more than 50% of the CREC isolates, highlighting the potential of these bacteria to develop colistin resistance. Therefore, active surveillance for *mcr*-9 is necessary to control further spread.

The diverse STs identified in our study indicated the genetic diversity of the ECC isolates, which was confirmed by phylogenetic analysis. Notably, the main clonally disseminated CREC strains, including ST418, ST171, ST97, and ST78, differed from the most widespread STs among global CREC isolates in 37 countries (ST114, ST93, ST90, and ST78) (Peirano et al., 2018) and from the primary epidemic ST120 clone in Henan Province, China (Liu et al., 2015). Of note, this is the first report of the spread of CREC ST418 clones during 2019–2021 in our hospital. These epidemic clones frequently co-carried *bla*NDM-1, *bla*SHV-12, and *bla*TEM-1B. Similarly, a study conducted in the Chinese cities Shenzhen and Dongguan indicated that ST418, which produces NDM-1 carbapenemase, is the main epidemic CREC strain (Jin et al., 2018), highlighting the necessity of active surveillance. To date, *E. hormaechei* ST182 was the main host for *bla*IMP-15 (Canada-Garcia et al., 2022); *E. hormaechei* ST50, ST66 and ST145 have been found to be VIM-producer in France (Emeraud et al., 2022); *E. hormaechei* ST90 isolates carrying the *bla*OXA-436 gene were suspected to be the source of the outbreak in Denmark (Raun-Petersen et al., 2022); And co-occurrence of *E. hormaechei* ST177 carrying *bla*NDM-1 and *bla*KPC-2 has been reported in China (Yang et al., 2018).

In Northeast USA, CREC ST171 carried *bla*KPC-3 on the IncFIA plasmid (Gomez-Simmonds et al., 2018), and stable uptake of the IncFIA plasmid helped ST171 to proliferate successfully throughout this area, as isolates lacking this plasmid were rare (Annavajhala et al., 2019). Although CREC ST78 clones have primarily been found in Northeast USA (Gomez-Simmonds et al., 2018), ST78 did not exhibit the same rapid clonal proliferation as ST171. Globally, CREC ST78 appears to be associated with various plasmid backbones, conferring its unique ability to acquire multidrug-resistance genes (Annavajhala et al., 2019). In Japan, ST78 isolates were found to carry *bla*IMP-1 on class 1 integrons encoded on multiple plasmids, such as IncHI2, IncW, and IncFIB (Aoki et al., 2018). Given the various plasmids in ST78, these strains may rapidly develop resistance to various antimicrobial agents under selective pressure. To the best of our knowledge, CREC ST252, ST1001, ST93, ST1373, ST32, ST88, ST136, ST233, ST316, ST459, ST461, ST994, ST1120, and ST1683 have not been previously reported.

Admission to the ICU was an independent risk factor of CREC acquisition in our study, which is in accordance with the finding of a study conducted in Southwest China (Tian et al., 2020), confirming the risk of ICU admission for CREC acquisition. Further analysis showed that ICU admission was also associated with the acquisition of endemic CREC-ST418 clones, which may be because endemic CREC ST418 was the main clone circulating in the ICU in our study. This was the first time we combined molecular typing with risk factor analysis.

This study had several limitations. First, although the strains were collected during 2013–2021, the sample size was relatively small and may not fully reflect the molecular epidemiology of CREC strains. Second, the study was performed in a single hospital, and the results cannot be generalized to other institutions.

In conclusion, *bla*NDM-1 and *bla*IMP-4 were the main *bla*CHβLs in our CREC isolates, and *bla*SHV-12 and *bla*TEM-1B were the major co-carried *bla*ESBLs. ST418 was the predominant ST, followed by ST171 and ST97, all of which displayed clonal dissemination. Plasmid replicons were diverse, with IncHI2, IncHI2A, and IncX3 being the most frequent replicons. As the wide spread of CREC, early detection and surveillance to prevent the emergence and further spread of antibiotic resistance are urgently needed.

## Data availability statement

The datasets presented in this study can be found in online repositories. The names of the repository/repositories and accession number(s) can be found in the article/Supplementary material.

## Ethics statement

The studies involving human participants were reviewed and approved by the Ethics Committee of Nanjing Drum Tower Hospital (Approval No. 2022-049-2). Written informed consent from the participants’ legal guardian/next of kin was not required to participate in this study in accordance with the national legislation and the institutional requirements.

## Author contributions

MH performed the bioinformatics analysis and wrote the manuscript. CL interpreted the data on the ARGs and plasmid replicons and revised the manuscript. HX sorted the data and helped with manuscript writing. JZ and YZ interpreted the data on the ARGs and plasmid replicons. CCL performed the statistical analysis. HS and XC designed the study and revised the manuscript. All authors read and approved the final manuscript.

## Footnotes

1
https://www.ncbi.nlm.nih.gov/bioproject/PRJNA868583

2
https://cge.food.dtu.dk/services/MLST/

3
https://cge.food.dtu.dk/services/ResFinder/

4
https://cge.food.dtu.dk/services/PlasmidFinder/
